# Increased Risk of Bleeding with Topical Metronidazole in a Postoperative Wound after Anal Fistula and Hemorrhoid Surgery: A Propensity Score-Matched Case–Control Study

**DOI:** 10.3390/clinpract12010017

**Published:** 2022-02-18

**Authors:** Pankaj Garg, Vipul D. Yagnik, Gurleen Kaur

**Affiliations:** 1 Department of General Surgery, Indus Super Specialty Hospital, Mohali 160052, India; 2 Department of Colorectal Surgery, Garg Fistula Research Institute, 1042, Sector-15, Panchkula 134113, India; 3 Department of General Surgery, Nishtha Surgical Hospital and Research Centre, Patan 384265, India; vipul.yagnik@nshrc.com; 4 Department of Pharmacology, Adesh Medical College and Hospital, Shahbad 136135, India; drgurleenkaur@amch.ac.in

**Keywords:** metronidazole, topical, bleeding, anal fistula, hemorrhoids, surgery

## Abstract

Background: Topical metronidazole (TM) is commonly used in many infective conditions and postoperative wounds including after anorectal surgery. TM was prescribed in patients operated for benign anorectal conditions (anal fistula and hemorrhoids) to hasten wound healing. After the initiation of this protocol, the incidence of postoperative wound bleeding seemed to increase. There are no data in the literature suggesting that topical metronidazole increases the risk of bleeding. Objective: Analysis of the association of TM with an increased risk of bleeding in postoperative anorectal wounds. Design: This was an observational and a retrospective study. Propensity score matching was performed. Setting: This study was conducted at a specialized center for anorectal disorders in postoperative patients suffering from anal fistula and hemorrhoids. Materials: The incidence of postoperative bleeding in the patients in whom TM was used (study group) was retrospectively compared with the patients operated one year before this period in whom TM was not used (control group). Sample size: There were 35 patients in the study group and 181 patients in the control group. Main outcome measures: The incidence of bleeding and the number of bleeding episodes were evaluated. Results: The incidence of bleeding was significantly higher in the study group as compared to the control group (8/35 (22.8%) vs. 8/181 (4.4%), respectively, *p* = 0.0011). In most cases, bleeding was controlled with conservative measures. The number of bleeding episodes was also significantly higher in the study group (14 vs. 11, respectively, *p* = 0.0001). The number of patients requiring operative intervention was also higher in the study group (2/35—5.7%) as compared to the control group (1/181—0.56%), but this was not statistically significant (*p* = 0.069). Conclusions: The study highlighted that application of topical metronidazole in postoperative anorectal wounds increased the risk of bleeding. Most of the bleeding episodes were controlled with conservative measures but they caused considerable patient anxiety and apprehension.

## 1. Introduction

Metronidazole is a 5-nitroimidazole derivative active as an antibiotic against anaerobic and aerobic bacteria and some protozoa [1]. Metronidazole is available as oral, parenteral, topical and intravaginal formulations. Topical metronidazole (TM) has been used for several reasons which include decreasing infection in wounds [1,2,3,4], promoting healing in anal fissures and surgical wounds [5,6,7,8,9], decreasing pain in postoperative wounds [8,9], etc.

As metronidazole is hydrophilic, only trace amounts penetrate healthy skin. Therefore, the side effects are limited to local skin reactions like burning and irritation [4]. Systemic toxicity on topical application is rare. There are no data in the literature suggesting that TM increases the risk of bleeding.

The reason for bleeding after TM application is uncertain though a possible reason could be local irritation. Regular TM application could inflame the capillaries and blood vessels repeatedly and lead to bleeding. However, this is speculative and the exact cause remains uncertain. The purpose of this study was to analyze the association of TM with the risk of bleeding in operative wounds.

## 2. Materials and Methods

### 2.1. Ethical Considerations

The study was commenced after receiving approval and clearance from the hospital ethics committee, Indus International Hospital–Institute Ethics Committee (IIH–IEC), Mohali, Punjab, India.

### 2.2. Study Design and Setting

At a specialized center for anorectal disorders, every patient operated for anal fistula and hemorrhoids between 1 October 2020 and 30 November 2020 was prescribed additional topical metronidazole (TM) to be applied to the postoperative wound on a daily basis. This was done to hasten postoperative anal wound healing as TM had been shown to heal chronic anal fissure wounds quite well [5]. Patients with coagulopathy or bleeding disorders were excluded from the study. TM was scheduled to be applied four times a day to the operative wound till it healed (for 6–8 weeks). TM was utilized in addition to topical xylocaine and sucralfate cream used for dressings. The patients who were operated for anal fistula and hemorrhoids between 1 October 2019 and 30 September 2020 were taken as the control group. In these patients, only topical xylocaine and sucralfate cream were used for dressings and no TM was used. The incidence of postoperative bleeding was retrospectively compared between these groups. In both groups, the patients on anti-platelet drugs or anticoagulants had their medication withheld five days before surgery and resumed five days after surgery.

As the difference between the number of patients between the study and the control group was quite high, therefore, propensity scoring and matching were also performed. Propensity scoring was performed for eight relevant parameters—age, sex, associated hemorrhoids, multiple tracts, horseshoe tracts, recurrent surgery, associated abscess and fistula complexity. The Garg classification was used for fistula classification as it has been shown to correlate better with fistula complexity [10].

### 2.3. Statistical Analysis

The categorical variables were compared by performing Fisher’s exact test or chi-squared analysis results. When the data were normally distributed, the continuous variables were analyzed using Student’s *t*-test when there were two samples. The significant cutoff point was set at *p* < 0.05. The SPSS Statistics 21.0 software was used for statistical analysis.

## 3. Results

**Figure 1:**
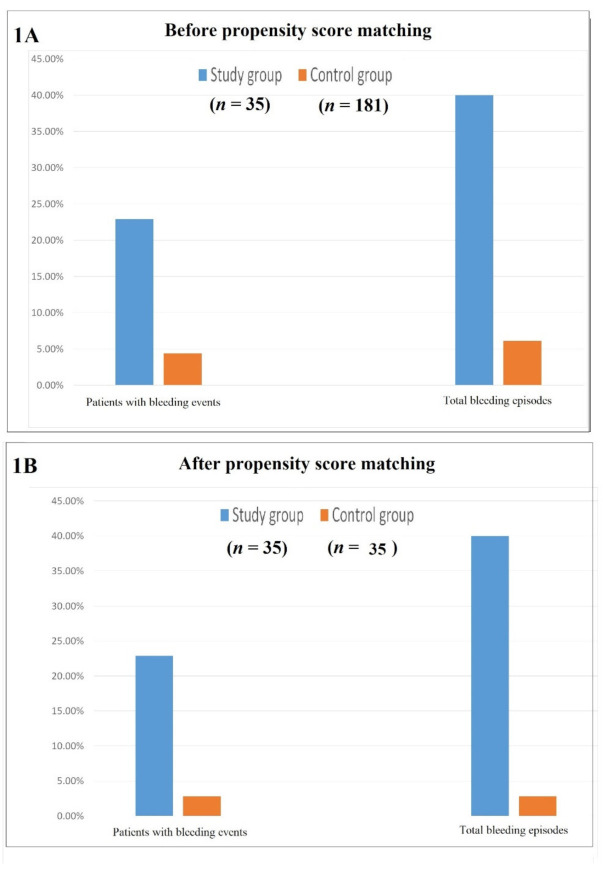
The incidence of patients with bleeding events and total bleeding episodes before propensity score matching (**A**) and after propensity score matching (**B**).

There were 35 patients in the study group and 181 patients in the control group. There was male preponderance, and the groups were comparable in terms of the age, sex ratio, fistula parameters and intake of antiplatelet or anticoagulant drugs (Table 1). However, associated hemorrhoids (along with fistula) was significantly higher in the study group (25.7% vs. 8.3%, *p* = 0.0006, Fisher’s exact test), although after propensity score matching, it became similar in both groups (25.7% vs. 22.9%, *p* = 1.0, Fisher’s exact test). The incidence of bleeding was significantly higher in the study group as compared to the control group (8/35 (22.8%) vs. 8/181 (4.4%), respectively, *p* = 0.0011, Fisher’s exact test) (Table 2) (Figure 1). Most bleeding events were controlled with conservative measures. The number of bleeding episodes was also significantly higher in the study group (14 vs. 11, respectively, *p* = 0.0001) (Table 2). The number of patients requiring operative intervention to control postoperative bleeding was also higher in the study group (2/35—5.7%) as compared to the control group (1/181—0.56%), but this was not statistically significant (*p* = 0.069, Fisher’s exact test) (Table 2). The number of patients with bleeding episodes and the total number of bleeding episodes remained significantly higher in the study group after matching with propensity scoring was performed (Table 2) (Figure 1). Expectedly, bleeding episodes caused considerable anxiety, distress and dissatisfaction among the patients.

## 4. Discussion

TM has been utilized and found quite beneficial in several conditions. TM is highly active against Gram-positive and -negative bacteria and is the treatment of choice for anaerobic infections [1]. TM has been used for the treatment of a wide range of wounds. TM helps reduce wound odor, decreases pain, decreases exudates, stops the progression of necrosis, reduces surrounding cellulitis and thus improves the wound [2]. TM is found to be very useful for the management of diabetic foot [3] and pressure ulcers [4].

TM is notably used in various anorectal disorders like anal fissures [5,6,11]. Pelta et al. used TM on “fissurotomy” wounds with good results [6]. However, oral metronidazole is not associated with faster healing in sphincterotomy [12]. TM has also been useful in fissures and ulcers associated with Crohn’s disease [7]. The postoperative wound in the perianal region is frequently in contact with fecal microflora and is at high risk of secondary infection. Carapeti et al. [9] and Nicholson and Armstrong [7] studied the effect of TM on post-hemorrhoidectomy wounds and found that healing is faster and less painful with TM. Finlay et al. [13] and Kalinksi et al. [14] reported a significant reduction in pain with TM on cutaneous ulcers and wounds. However, the mechanism of action of TM in reducing postoperative pain in unknown [15]. Karapolat used TM for cases of acute fissure-in-ano and showed that it is safe and effective for healing [16].

The essential factors to consider in the healing of perianal wounds are inflammation and infection. The infection causes a rapid increase in exudate production that needs to be effectively managed to protect the surrounding skin from maceration and excoriation and is also associated with delayed wound healing [17]. Metronidazole can favorably influence wound healing, promote epithelialization and decrease the tissue repair time [8,18,19,20]. Grekova et al. [21] found that patients with chronic anal fissure and anaerobic bacteria colonization benefited from TM treatment; they experienced a faster relief of pain and sphincter spasms, as well as enhanced fissure healing (healing rate: 95.6%). TM is also safe and effective for the local treatment of malodorous neoplastic lesions [22]. The deodorizing effect is due to eradication of anaerobic organisms [15]. TM has an excellent safety profile, with local skin irritation and burning being the major side effects. No systemic toxicity has been observed. Owing to these properties, as mentioned earlier, we included topical metronidazole to reduce infection, pain and hasten local wound healing after anorectal surgery.

There is no literature available discussing the incidence of bleeding after the use of topical metronidazole. The reasons for increased incidence of bleeding in the study group in the present study could be the greater frequency of application of TM. In the previously published studies, TM was applied to the wound once or twice a day, whereas in the present study, TM was applied four times daily. In some published studies, TM was applied to a closed wound [7], whereas in the present study, it was applied to open wounds. These could be few reasons for higher incidence of bleeding in this study. It is pertinent to point out that the increased risk of bleeding with TN was observed only in postoperative wounds and there is no evidence to extrapolate this risk to chronic wounds like diabetic ulcers, chronic anal fissures, etc.

The increased risk of hemorrhage in anticoagulated patients due to the interaction of oral metronidazole and warfarin is well-documented [23]. Metronidazole delays metabolism of warfarin and enhances the anticoagulant effect; this increases the likelihood of bleeding complications and is manifested in a raised INR. This interaction of oral metronidazole and warfarin is not due to CYP2C9 inhibition [24]. Impaired production of vitamin K by gastrointestinal flora, alterations brought about by oral metronidazole may also be a factor, though this is likely only in prolonged antibiotic usage with no oral diet. However, these mechanisms apply only to the high serum levels achieved after intravenous or oral dosing. Sampaio CPP et al. showed that in experimental rats, the serum concentration of metronidazole following topical application to a skin wound was between 0.001% and 0.002% and was uninfluenced by the concentration, duration and surface area (wound extent) on which TM was applied [25]. This characterizes an extremely low level of metronidazole in the bloodstream following topical application and does not appear to be responsible for the increased hemorrhagic risk.

The results of the study corroborate our clinical suspicion of the association of topical metronidazole (TM) with postoperative wound bleeding. To our knowledge, this is the first study which establishes this association. Any bleeding from a postoperative wound causes immense anxiety and frustration in the mind of the patient as well as of the surgeon. Therefore, the increased risk of bleeding with TM is a matter of substantial concern.

Our study is limited by its retrospective nature and sample size. Though unlikely, we could have ruled out systemic absorption effects by tracking the INR in the study group. Furthermore, topical metronidazole was applied in conjunction with xylocaine in all the patients. This could have introduced confounding bias since the vasodilation induced by xylocaine rather than by metronidazole could have increased the risk of bleeding. However, xylocaine was used in both the study and the control groups. Therefore, the chances of this bias are extremely remote.

## 5. Conclusions

To our knowledge, this is the first study in which topical metronidazole is shown to be associated with an increased risk of bleeding from anorectal surgical wounds. Our results raise a warning flag to create awareness among clinicians of this undocumented surgical complication. Further research is warranted to quantify the risk and identify the underlying mechanisms.

## Figures and Tables

**Table 1:** Demographic profile and disease parameters of both groups of patients.

	Before PS Matching			After PS Matching		
	Study Group (*n* = 35)	Control Group (*n* = 181)	Test of Significance (*p* < 0.05 = Significant)	Study Group (*n* = 35)	Control Group (*n* = 35)	Test of Significance (*p* < 0.05 = Significant)
Surgery performed (number of patients)	Anal fistula—25 Anal fistula + Hemorrhoids—9 Hemorrhoids—1	Anal fistula—166 Anal fistula + Hemorrhoids—15		Anal fistula—25 Anal fistula + Hemorrhoids—9 Hemorrhoids—1	Anal fistula—27 Anal fistula + Hemorrhoids—8	
Age (years) (mean ± SD)	40.1 ± 10.7	39.2 ± 12.5	^††^ *p* = 0.94	40.1 ± 10.7	36.83 ± 9.79	^††^ *p* = 0.15
M/F ratio	29/6	160/21	^†^ *p* = 0.41	29/6	29/6	^†^ *p* = 1
Associated hemorrhoids	9 (25.7%)	15 (8.3%)	^†^ *p* = 0.006	9 (25.7%)	8 (22.9%)	^†^ *p* = 1
Recurrent fistula	12 (34.3%)	88 (48.6%)	^†^ *p* = 0.14	12 (34.3%)	12 (34.3%)	^†^ *p* = 1
Associated abscess	8 (22.9%)	50 (27.6%)	^†^ *p* = 0.67	8 (22.9%)	9 (25.7%)	^†^ *p* = 1
Horseshoe tracts	6 (17.1%)	53 (29.3%)	^†^ *p* = 0.15	6 (17.1%)	5 (14.3%)	^†^ *p* = 1
Multiple tracts	25 (71.4%)	140 (77.3%)	^†^ *p* = 0.51	25 (71.4%)	25 (71.4%)	^†^ *p* = 1
Complexity of the fistula Simple/complex	18/16	96/85	^†^ *p* = 1.0	18/16	19/16	^†^ *p* = 1
Number of patients on antiplatelet/anticoagulant drugs (stopped 5 days prior to surgery)	1 (2.9%)	7 (3.9%)	^†^ *p* = 1.0	1 (2.9%)	1 (2.9%)	^†^ *p* = 1.0

Note: *n* = number, M: male, F: female, PS—propensity score, simple fistula—Garg grade I–II, complex fistula—Garg grade III–V, ^†^ Fisher’s exact test, ^††^ Student’s *t*-test. Study group: patients with topical metronidazole dressings. Control group: patients with no topical metronidazole dressings.

**Table 2:** Comparison of bleeding incidence in both groups of patients.

	Before PS Matching			After PS Matching		
	Study Group (*n* = 35)	Control Group (*n* = 181)	Test of Significance	Study Group (*n* = 35)	Control Group (*n* = 35)	Test of Significance
Patients with bleeding events	8 (22.9%)	8 (4.4%)	^†^ *p* = 0.0011 *	8 (22.9%)	1 (2.8%)	^†^ *p* = 0.027 *
Total bleeding episodes	14 (40%)	11 (6.1%)	*p* = 0.0001 *	14 (40%)	1 (2.8%)	^†^ *p* = 0.0002 *
Patients requiring an operative intervention to control bleeding	2 (5.7%)	1 (0.56%)	^†^ *p* = 0.069	2 (5.7%)	0	^†^ *p* = 0.49

Antiplatelet and anticoagulant drugs were stopped five days before surgery and resumed five days after surgery. Note: * significant (*p* < 0.05 = significant), ^†^ Fisher’s exact test.

## Data Availability

All the data are available from the corresponding author.
